# Treatment of Laryngoceles: What Is the Progress over the Last Two Decades?

**DOI:** 10.1155/2014/819453

**Published:** 2014-03-06

**Authors:** Karol Zelenik, Lucia Stanikova, Katarina Smatanova, Michal Cerny, Pavel Kominek

**Affiliations:** ^1^Department of Otorhinolaryngology, University Hospital Ostrava, 17. listopadu 1790, 708 52 Ostrava, Czech Republic; ^2^Department of Otorhinolaryngology and Head and Neck Surgery, Faculty of Medicine in Hradec Kralove,University Hospital Hradec Kralove, Charles University in Prague, Sokolska 581, 50 005 Hradec Kralove, Czech Republic

## Abstract

*Objectives*. To review surgical techniques used in the treatment of laryngoceles over the last two decades and point out developments and trends. *Materials and Methods*. PubMed, the Cochrane Library, and the JBI Library of Systematic Reviews were searched using the term “laryngocele.” Demographic data, type of laryngocele, presence of a laryngopyocele, type of treatment and need for a tracheotomy were assessed. *Results*. Overall, data on 86 patients were analyzed, culled from 50 articles, of which 41 were case reports and 9 were case series. No single systematic review or meta-analysis or randomized controlled trial has been published on the topic. Altogether, 71 laryngoceles in 63 patients met the criteria for further analysis focusing on surgical treatment. An external approach was selected in 25/29 (86.2%) cases of combined laryngoceles. Microlaryngoscopic resection using a CO_2_ laser was performed in three cases and endoscopic robotic surgery in one case. The majority of patients with an internal laryngocele, 31/42 (73.8%), were treated using the microlaryngoscopy approach. *Conclusions*. Microlaryngoscopy involving the use of a CO_2_ laser has become the main therapeutic procedure for the treatment of internal laryngoceles during the past 20 years. An external approach still remains the main therapeutic approach for the treatment of combined laryngoceles.

## 1. Introduction

A laryngocele is an abnormal dilation of the laryngeal saccule that extends upward within the false vocal fold, is filled with air, and is in communication with the laryngeal lumen [1, 2]. The term laryngocele should be used only when the lesion is symptomatic, palpable, or visible during laryngoscopy or when it is found to extend above the upper border of thyroid cartilage [1].

There are currently three main theories regarding the etiology of laryngoceles: congenital factors, increased laryngeal pressure, and mechanical obstruction [3, 4]. It has been stated that prolonged periods of increased laryngeal pressure (e.g., in glass blowers and wind instrument players) could result in gradual dilation of the saccule [5]. The use of a laryngeal mask during general anaesthesia can have the same effect [6]. Moreover, mechanical obstruction of the ventricle as a result of acquired laryngeal disease (carcinoma, chondroma, amyloidosis, and others) can cause increased intraventricular pressure and promote dilatation of the saccule [3, 7–10].

**Figure 1:**
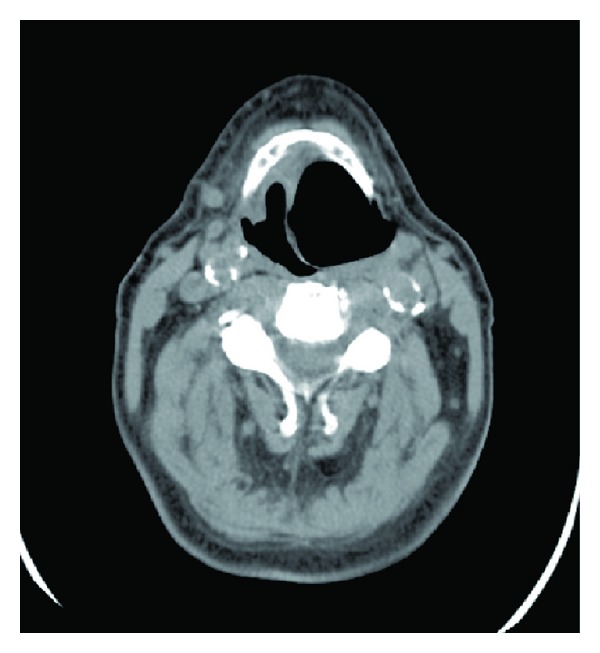
Right internal laryngocele.

**Figure 2:**
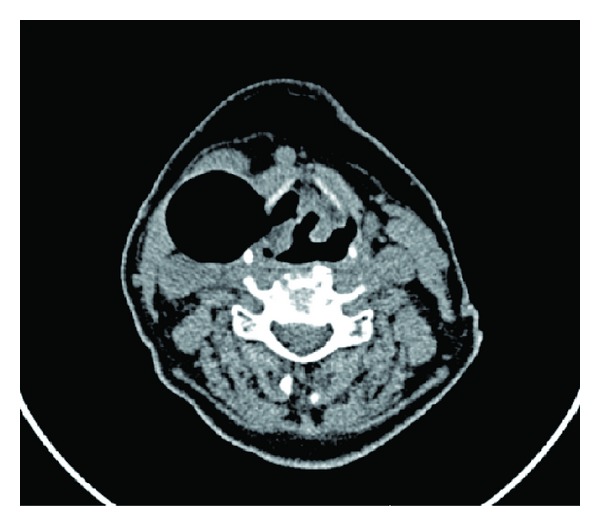
Left combined laryngocele.

Laryngoceles are categorized as internal (Figure 1) or combined (Figure 2) [11]. The formerly used classification into internal, external, and combined laryngoceles is being abandoned because purely external laryngoceles cannot exist, as laryngoceles originate at the laryngeal saccule. An internal laryngocele is confined within the false vocal fold, medial to the thyrohyoid membrane. A combined laryngocele extends upward and protrudes through the thyrohyoid membrane to the neck [11]. If the neck of the laryngocele becomes obstructed (causes of which include tumours and chronic inflammation of the larynx), the mucus produced by the mucous glands of the lining epithelium can accumulate, leading to a laryngomucocele. When it is infected, a laryngopyocele forms [12].

Laryngoceles are uncommon entities and currently there is no consensus regarding their surgical treatment. Various modalities of treatment have been utilized [11, 13–18]. An external approach, the traditional treatment, is still being advocated by some authors [11, 13] However, endoscopic management of laryngoceles has gained popularity during the last two decades, following the advent of microlaryngoscopic surgery and CO_2_ lasers [14, 17, 18].

The objective of this paper is to review the existing body of literature on the subject, find out which surgical techniques have been used for the treatment of laryngoceles within the last two decades, and point out developments and trends. To our knowledge, a review article summarizing laryngocele treatment has not been published to date.

## 2. Materials and Methods

PubMed, the Cochrane Library, and the JBI Library of Systematic Reviews were searched using the term “laryngocele” to identify articles published on the topic within the period 1994–2013. All articles were reviewed by two independent reviewers and only those written in the English language, dealing with adult patients and with a stated therapeutic approach, were selected for the study. Data on patients who did not undergo surgery and on those with laryngoceles and associated tumors which required specific surgical treatments were excluded from further analysis of surgical methods.

Demographic data (sex, age), type of laryngocele (internal, combined, unilateral, and bilateral), presence of a laryngopyocele, type of treatment, the inclusion of a tracheotomy as part of the treatment, and recurrence were assessed.

Laryngoceles formerly claimed to be external were reclassified as combined for the purpose of this study, because purely external laryngoceles cannot exist, as laryngoceles originate at the laryngeal saccule [11].

The following types of external surgery were ascertained to have been performed: transthyrohyoid membrane approach, thyrotomy with resection of the upper 1/3 of thyroid cartilage, and V-shaped thyrotomy. The following types of endolaryngeal surgery were ascertained to have been performed: microlaryngoscopy using a CO_2_ laser or cold instruments, marsupialization, and robotic surgery.

Descriptive statistics using Microsoft Excel were used to analyze the results.

## 3. Results

Using the term “laryngocele” a total of 123 articles published within the period 1994–2013 were found on PubMed. Searches of the Cochrane Library and the JBI Library of Systematic Reviews did not yield any systematic reviews or meta-analyses on the topic. Overall, data on 86 patients culled from 50 articles [6, 8–56], of which 41 were case reports and 9 were case series, met the inclusion criteria. No single systematic review or meta-analysis or randomized controlled trial had been published on the topic.

Data on up to 23 patients were excluded from further analysis focusing on surgical treatment, because 15 of them did not undergo surgery and 8 had an associated tumor that required a specific surgical treatment.

Overall, data on 63 patients were included for further analysis focusing on surgical treatment. Demographic data and results are summarized in Tables 1 and 2. Of the 63 patients, 35 (55.6%) were male and 28 (44.4%) female. Average age of the patients was 50.75 years. 55 had unilateral laryngoceles and 8 had bilateral laryngoceles, so surgical treatment of a total of 71 laryngoceles, 42 (59.2%) internal, and 29 (40.8%) combined was analyzed. Laryngopyocele was listed in 12 (16.9%) cases. Tracheotomy as a part of the surgery was done in 11/63 (17.5%) patients. In 6/63 (9.5%) patients a tracheotomy was performed as an urgent surgery to preempt the risk of suffocation.

An external approach was selected in 25/29 (86.2%) cases of combined laryngoceles. Surgical procedures included the transthyrohyoid membrane approach (an approach that does not involve resection of the thyroid cartilage) in 17/29 (58.6%) cases, thyrotomy with resection of the upper 1/3 of thyroid cartilage in four cases, and V-shaped thyrotomy in four cases. Microlaryngoscopic resection using a CO_2_ laser was performed in three cases and endoscopic robotic surgery in one case.

The majority of patients with internal laryngoceles, 31/42 (73.8%), were treated using the microlaryngoscopy approach. Resection using a CO_2_ laser was done in 24 cases, resection using cold instruments in two cases and marsupialization in five cases. An external approach was selected in nine cases, among which were one transthyrohyoid membrane approach and eight V-shaped thyrotomies.

## 4. Discussion

The incidence of laryngocele is estimated to be 1 per 2.5 million of the population per year [57] and laryngoceles have been reported to be five times more frequent in men, with a peak incidence in the sixth decade of life [3, 58]. In contrast, our review had a male-to-female ratio of 1.25 : 1 and peek incidence in the fifth decade. These results are similar to the results of Devesa et al. [18], who reported a male-to-female ratio of 7 : 5 and a peak incidence in the fifth decade in their series. 87% of the laryngoceles in our data were unilateral, which is consistent with previously published data [18, 58].

Laryngocele is a rare condition that presents a surgical dilemma. As a result, many types of surgery have been used in its treatment. Excision of both types, combined and internal, was traditionally done using an external approach [13]. However, with the advent of microlaryngoscopic surgery and the CO_2_ laser during the last two decades, the endolaryngeal technique has gained popularity and many of the authors reviewed have begun to use this technique for the treatment of internal laryngoceles [17, 18]. Moreover, some authors have begun to use a microlaryngoscopy technique for the treatment of combined laryngoceles as well [18]. On the other hand, the external approach is still being advocated by some authors [11, 13].

When discussing surgical procedures used for the treatment of laryngoceles during the last 20 years, which was the main goal of our review, it is necessary to consider combined and internal laryngoceles separately.

Most of the patients with combined laryngoceles (86.2%) were treated using an external approach. The reported advantages of external approaches are good exposure of the laryngocele, a more precise procedure and a low recurrence rate. Disadvantages are skin scarring, higher morbidity, longer duration of surgery, longer hospitalization period, and higher costs [11]. Three types of external procedures have been used during the past 20 years—the transthyrohyoid membrane approach, thyrotomy with resection of the upper 1/3 of the thyroid cartilage, and V-shaped thyrotomy [11, 13, 27, 30, 35, 46, 50]. The transthyrohyoid membrane approach was the one used most often, in 68% of the cases treated using external techniques. The advantage of this procedure when compared with the other two is that no resection of the thyroid cartilage is done. The disadvantage is limited exposure of the paraglottic space. Thyrotomy with resection of the upper 1/3 of the thyroid cartilage and V-shaped thyrotomy were used in a minority of patients [11, 13, 50]. As part of these techniques, a portion of the thyroid cartilage is resected. This enables better exposure ofparaglottic space [11].

Interestingly, four patients with combined laryngoceles were treated using the endolaryngeal approach [18]. Devesa et al. reported on 12 patients with laryngoceles treated using the microlaryngoscopic approach and a CO_2_ laser, of whom three had combined laryngoceles. The authors describe how they deal with the external part of the combined laryngoceles. Once the internal component is isolated, any lateral external component can be drawn into the laryngeal lumen gradually by a mixture of laser mobilization, traction, and blunt microsurgical dissection. If the bulk of the mobilized laryngocele becomes too large for ease of handling endoscopically, it is a simple matter to excise the more medial and superior component and then to continue with the remainder [18].

The first endolaryngeal resection of a combined laryngocele using robotic surgery was reported in 2013 [16]. According to the authors, this technique seems to have several advantages when compared with microlaryngoscopy. For instance, optics are placed in the oral cavity, thus allowing closer, angulated vision of the surgical field. In addition, rather than using traditional laryngoscopes, instruments are introduced through mouth gags, which offer a wider view and range of motion. Furthermore, miniaturized, angulated, and “tremor-filtered” robotic instruments with “wristed-tips” enable one to reach far lateral (hidden) areas [16].

An interesting procedure was described by Szwarc and Kashima in 1997 [14]. The authors put their patient with a combined laryngocele on intravenous antibiotics for two weeks. As a supportive treatment they used “warm throat irrigations” and prohibited smoking. As a result, the external part of the combined laryngocele vanished and the internal part was then resected using a CO_2_ laser via the microlaryngoscopy approach [14].

The majority of internal laryngoceles in our review, 31 out of 42 (73.8%), were treated using the endolaryngeal (microlaryngoscopy) approach. Resection using a CO_2_ laser is currently the preferred and most frequently used type of surgery for internal laryngoceles. It was performed in 24 cases. This technique is considered by many authors to be a quick, precise, and safe alternative to an external approach excision, with fewer complications than its external counterparts, resulting in speedier rehabilitation of both the patient and his or her voice [14, 17, 18, 21, 38, 41]. Moreover Devesa et al. advocate using this technique for the treatment of combined laryngoceles [18].

On the other hand, the endolaryngeal management of laryngoceles has the disadvantages of providing limited surgical exposure, causing endolaryngeal scarring and requiring experience with special instruments [11]. Furthermore, the probability of incomplete resection of large laryngoceles limits use of the endoscopic approach [11]. However, the last seems not to be of clinical significance, since no recurrence has been reported to date following the use of endolaryngeal techniques.

Two patients with internal laryngoceles were operated on via microlaryngoscopy using cold instruments [9, 32]. A risk of more severe bleeding is associated with this type of surgery, which prolongs the duration of surgery as well. A decreased incidence of bleeding is the main advantage of the introduction of a CO_2_ laser into microlaryngeal surgery, which is why most microlaryngoscopy resections are currently done using a CO_2_ laser [17].

Marsupialization of the laryngocele was performed in five cases. This technique entails a longer healing period and the risk of recurrence, since during the process of healing mucosa can form a new mucocele or cyst over the residual sack [18].

An external approach was selected in nine (26.2%) patients with internal laryngoceles, of which a transthyrohyoid membrane approach was adopted in one case and V-shaped thyrotomies in eight cases. This number seems to be quite high, given that the endoscopic approach has been advocated as the appropriate treatment for internal laryngoceles. However, after detailed examination, the following fact came to light. The transthyrohyoid membrane approach was adopted by Myssiorek et al. in 2001 in one case [13] and the performance of V-shaped thyrotomies was reported by Thomé et al. in 2000 in eight cases [11], which means that no single external approach has been reported in the treatment of internal laryngoceles during the past 12 years.

Tracheotomies were part of the surgery done on 11/63 (17.5%) patients. In six (9.5%) patients, a tracheotomy was done as an urgent surgery to preempt the risk of suffocation. This means that almost 10% of laryngoceles present as emergencies, something that is important to keep in mind. Moreover, two fatal cases of laryngocele were reported. In one case a 55-year-old woman with an obstructing combined laryngocele refused acute treatment and hospitalization and died suddenly a few minutes after leaving the hospital [53]. The other patient, a 70-year-old man, died during the night while waiting for surgery scheduled for the next day [51]. In light of the above mentioned cases, acute resection of the laryngocele or tracheotomy should be done as a “lege artis” action in patients at risk of suffocation, particularly in patients with a laryngocele having an extensive internal part.

## 5. Conclusion

The traditional treatment of a laryngocele was excision using an external approach. Advances in endoscopic techniques and the development and further applications of the laser in surgery have generated a new philosophy in the management of laryngeal diseases. Microlaryngoscopy with use of a CO_2_ laser has become the main therapeutic procedure for the treatment of internal laryngoceles during the past 20 years. Moreover, microlaryngoscopy has been used in all reported cases of internal laryngoceles during the last 12 years. An external approach still remains the main therapeutic approach for the treatment of combined laryngoceles. But endolaryngeal surgery has begun to be used too. Robotic surgery seems a promising method in the treatment of combined laryngoceles, but its potential advantages have yet to be proved.

## Figures and Tables

**Table 1:** Summary of demographic data and basic results.

Number of patients	63
Sex	Men = 35 (55.6%), women = 28 (44.4%)
Average age	50.75 years
Number of unilateral and bilateral laryngoceles	Unilateral = 55 (87.3%), bilateral = 8 (12.7%)
Type of laryngocele	Internal = 42 (59.2%), combined = 29 (40.8%)
Laryngopyocele	12 (16.9%)
Tracheotomy	11 patients (17.5%)
Recurrence	None reported

**Table 2:** Surgical treatment of 63 combined and internal laryngoceles.

Type of laryngocele	Treatment	Specific type of surgery
Combined = 29	External = 25	Transthyrohyoid membrane approach = 17 Thyrotomy with resection of the upper 1/3 thyroid cartilage = 4 V-shaped thyrotomy = 4
	Endolaryngeal = 4	Microlaryngoscopic CO_2_ laser resection = 3 Endoscopic robotic surgery = 1

Internal = 42	External = 9	Transthyrohyoid membrane approach = 1 V-shaped thyrotomy = 8
	Endolaryngeal = 31	Microlaryngoscopic CO_2_ laser resection = 24 Microlaryngoscopic resection using cold instruments = 2 Marsupialization = 5
